# Supplementary material for: Adaptive mechanisms facilitate robust performance in noise and in reverberation in an auditory categorization model
Source: Commun Biol. 2023 May 2;6:456. doi: 10.1038/s42003-023-04816-z (PMC10154343; doi:10.1038/s42003-023-04816-z)
Supplement: Supplementary file 2 — Supplementary Information [file 42003_2023_4816_MOESM2_ESM.pdf]

Adaptive mechanisms facilitate robust performance in noise and in reverberation in an auditory categorization model

Satyabrata Parida[1, 2], Shi Tong Liu[3], Srivatsun Sadagopan[1,2,3,4,*]

[1] Department of Neurobiology, University of Pittsburgh, Pittsburgh PA, USA
[2] Centre for the Neural Basis of Cognition, University of Pittsburgh, Pittsburgh PA, USA
[3] Department of Bioengineering, University of Pittsburgh, Pittsburgh PA, USA
[4] Department of Communication Science and Disorders, University of Pittsburgh, Pittsburgh PA, USA

*Corresponding author

Srivatsun Sadagopan, PhD

3501 5th Avenue,

BST-3 10021

Pittsburgh, PA 15261.

Phone: 412-624-8920

Email: vatsun@pitt.edu

Supplementary Figure S1. Temporal and spectral characterization of reverberant conditions used in the study.

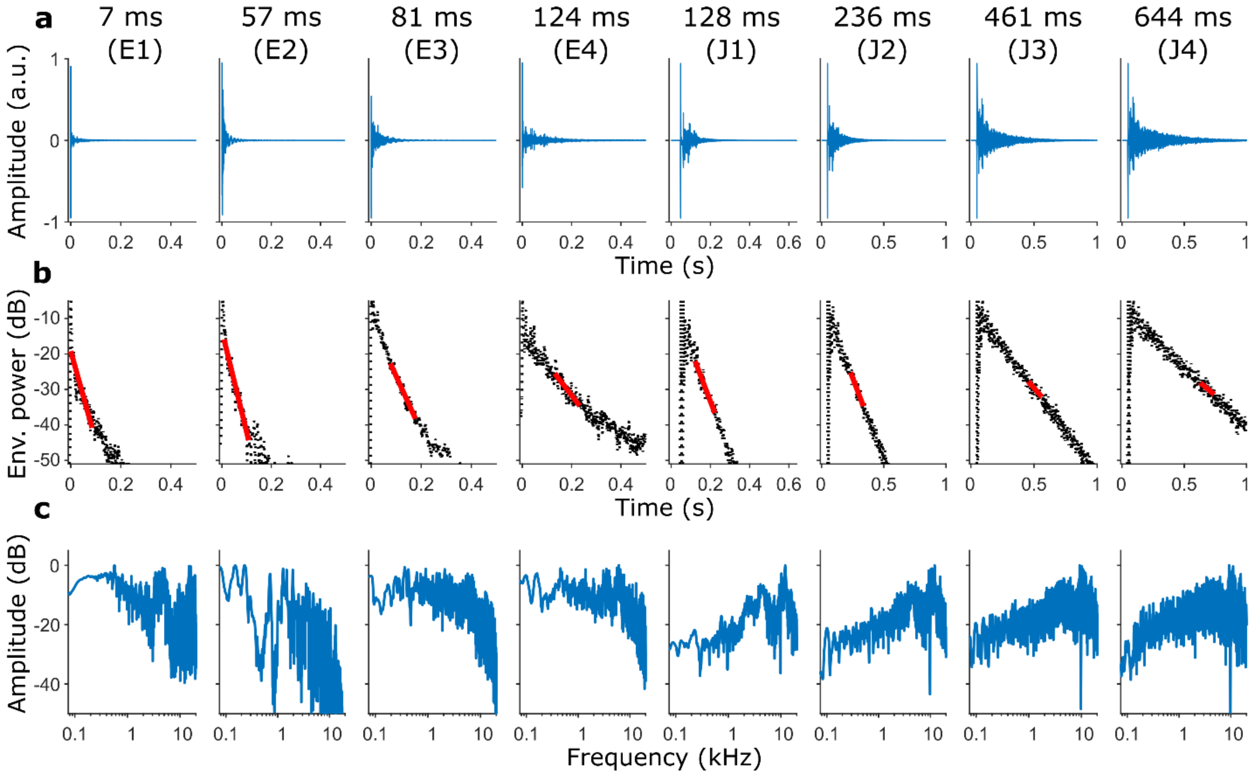

a Impulse responses of the eight reverberant conditions. **b** Black dotted line represents the envelope power decay as a function of time. For each impulse response, T30 was estimated by first considering a 100-ms window centered at the time when envelope power dropped below -30 dB, and then fitting a linear-regression line (red, time versus dB-power) and estimating T30 as the time when the linear line crossed -30 dB. **c** The Fourier spectrum of the impulse responses in **a**.

Supplementary Figure S2. For marmoset call types, properties of FDs were not systematically different across training conditions except for longer duration and lower threshold in noise training.

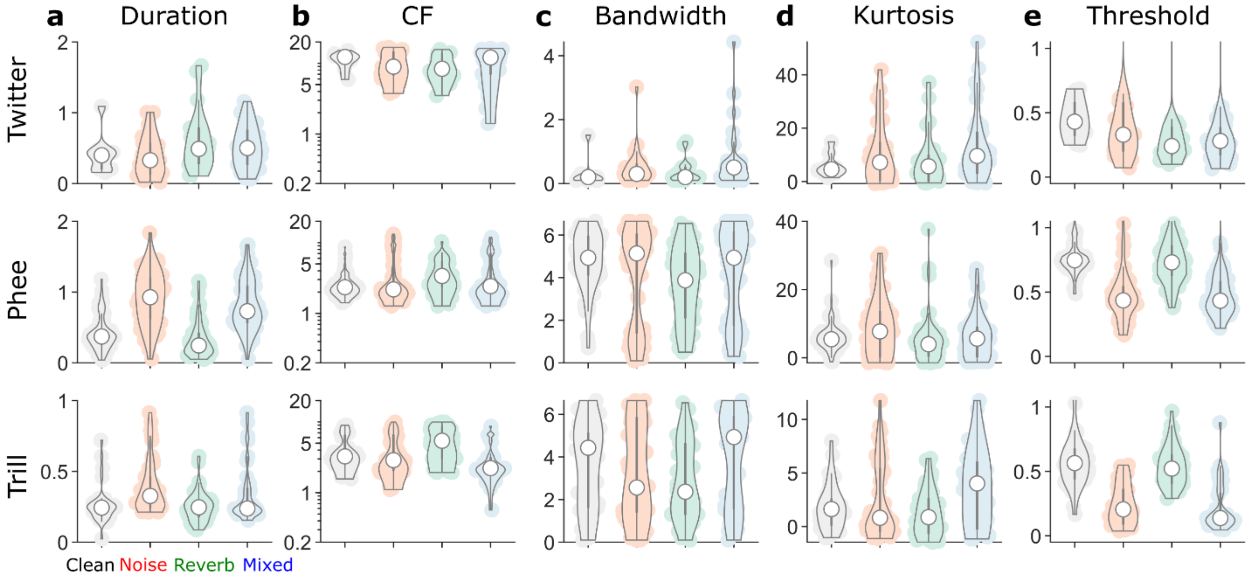

a-e Distributions of FD properties including duration (in seconds) (**a**), center frequency (kHz) (**b**), bandwidth (octaves) (**c**), reduced kurtosis (**d**), and threshold (**e**) of the FD spectrotemporal receptive field for three different marmoset call types (rows). Distributions were constructed using five different model instantiations for each call type. Statistics are reported in Supplementary Table S2.

Supplementary Figure S3. For guinea pig call types, the properties of FDs were not systematically different across training conditions.

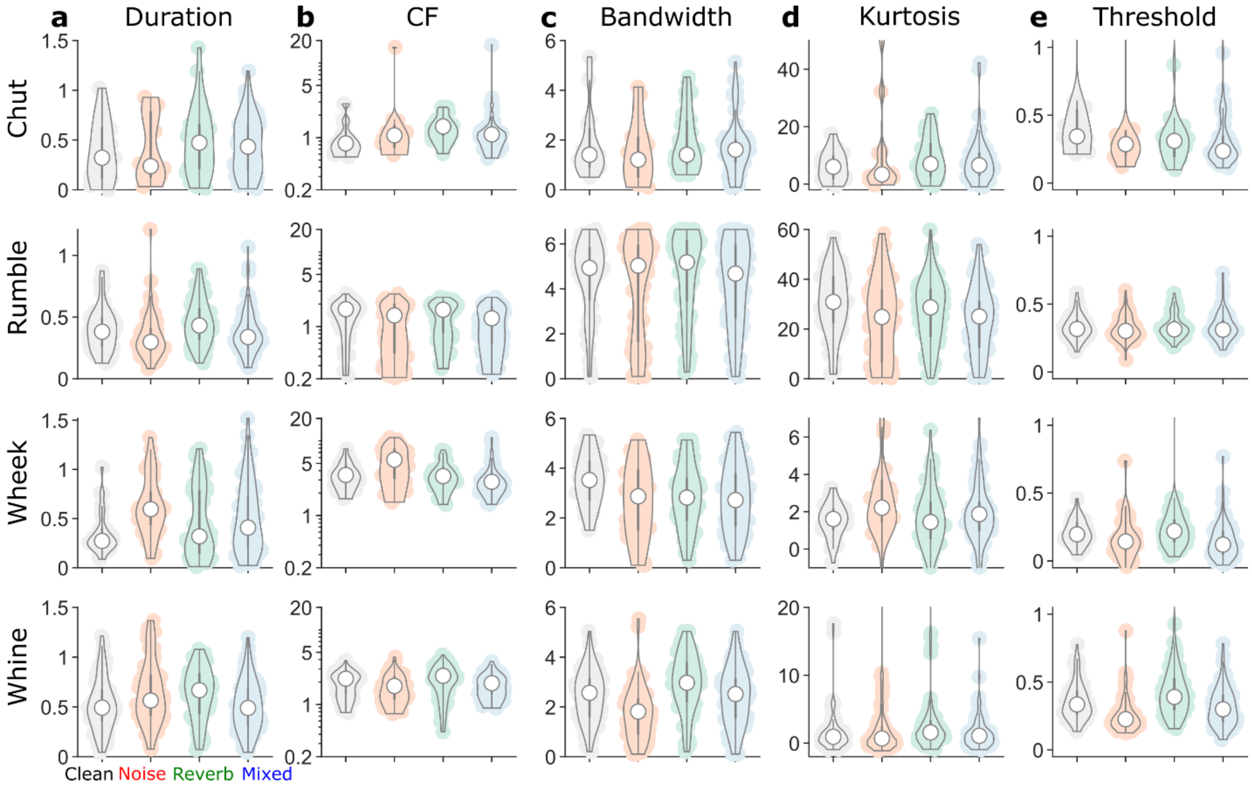

Same format as Supplementary Figure S2, but for guinea pig call types. Statistics are reported in Supplementary Table S3.

Supplementary Figure S4: The ideal-observer WTA d’ was transformed using a two-parameter logistic function to adjust for stimulus-independent factors that may affect behavior.

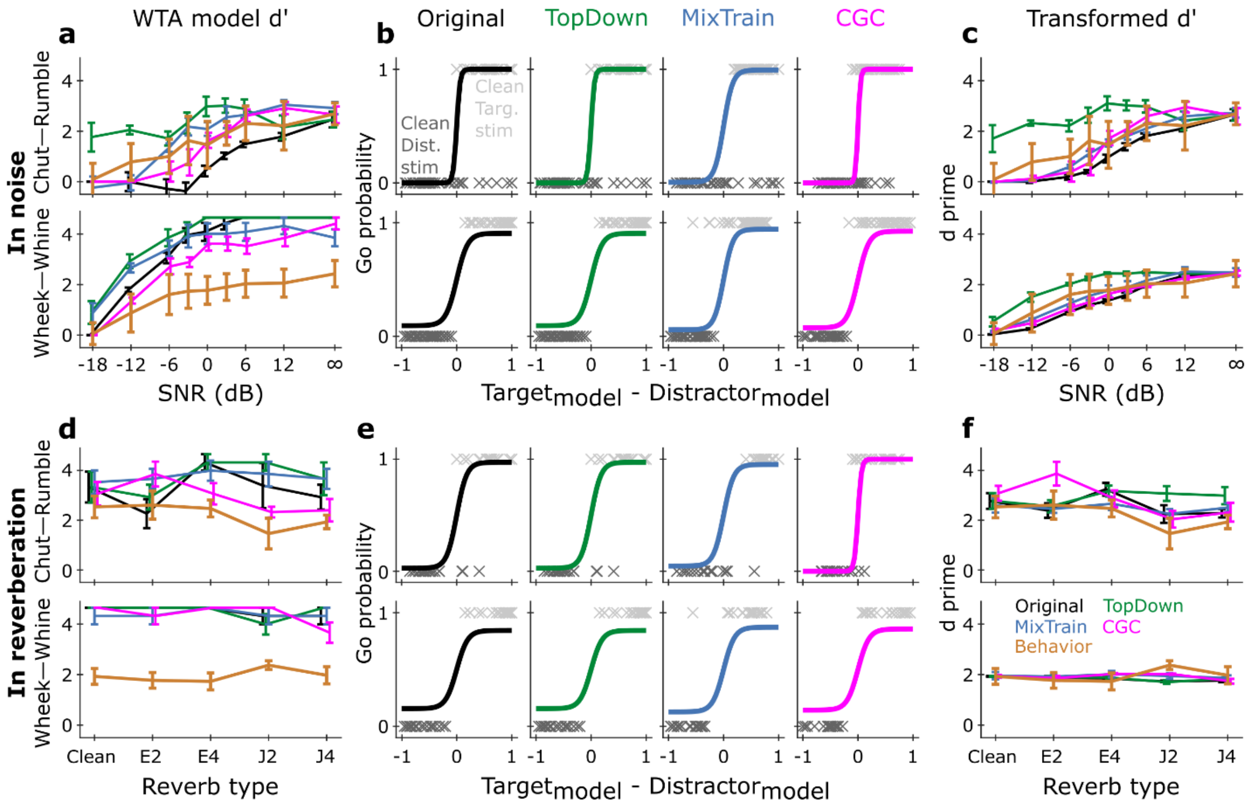

a The performance of the WTA ideal-observer model in noisy conditions for the chut-vs-rumble (top, target=chut) and wheek-vs-whine (bottom, target=wheek) go/no-go tasks. Line colors correspond to four different models and behavior (see legend text in **f**). **b** Dark (light) crosses correspond to WTA output for clean target (distractor) stimuli. A logistic function was learned to transform WTA output to go-rate for each mechanism (columns) by matching model d’ with behavioral d’ in the clean condition for five different instantiations of each model. **c** Performances of different models and animal behavior after applying the learned transformation at each SNR. **d-f** Same format as **(a-c)** but for four reverberation conditions. Logistic transformations **(e)** were learned to match WTA model d’ with behavior d’ **(d)** in the clean condition and were applied to transform model performances in different reverberant conditions **(f)**.

CGC, contrast gain control; WTA, winner-take-all

Supplementary Table S1: Effect of training relative to baseline model

	Noisy training		Reverberation training		Mixed training	
	Marmoset	Guinea pig	Marmoset	Guinea pig	Marmoset	Guinea pig
Test in noise	$\chi^2(3) = 145.0$<br>$p < 2.2 \times 10^{-16}$	$\chi^2(4) = 64.5$<br>$p = 3.2 \times 10^{-13}$	$\chi^2(3) = 85.7$<br>$p < 2.2 \times 10^{-16}$	$\chi^2(4) = 5.6$<br>$p = 0.23$	$\chi^2(3) = 173.1$<br>$p < 2.2 \times 10^{-16}$	$\chi^2(4) = 42.6$<br>$p = 1.2 \times 10^{-8}$
Test in Reverb	$\chi^2(3) = 81.1$<br>$p < 2.2 \times 10^{-16}$	$\chi^2(4) = 28.8$<br>$p = 8.4 \times 10^{-6}$	$\chi^2(3) = 204.4$<br>$p < 2.2 \times 10^{-16}$	$\chi^2(4) = 71.0$<br>$p = 1.4 \times 10^{-14}$	$\chi^2(3) = 219.2$<br>$p < 2.2 \times 10^{-16}$	$\chi^2(4) = 98.6$<br>$p < 2.2 \times 10^{-16}$

Test performance was better for models with same-condition training than for models with across-condition training (as indicated by χ^2 values). Moreover, models with mixed training (i.e., trained with clean, noisy, and reverberant calls) generally outperformed models trained only in either noisy or reverberant condition.

Supplementary Table S2: Effect of noisy and reverberation training on FD properties for marmoset calls.

Training	Duration	CF	Bandwidth	Kurtosis	Threshold
Noise	$\eta_\mathrm{p}^2 = 0.28$ $F_{1,319} = 122.5$ $p < 2.2 \times 10^{-16}$	$\eta_\mathrm{p}^2 = 0$ $F_{1,319} = 0.05$ $p = 0.83$	$\eta_\mathrm{p}^2 = 0$ $F_{1,319} = 1.4$ $p = 0.24$	$\eta_p^2 = 0.03$ $F_{1,319} = 11.0$ $p = 9.9 \times 10^{-4}$	$\eta_p^2 = 0.22$ $F_{1,319} = 89.7$ $p < 2.2 \times 10^{-16}$
Reverb	$\eta_\mathrm{p}^2 = 0$ $F_{1,357} = 1.3$ $p = 0.26$	$\eta_\mathrm{p}^2 = 0.04$ $F_{1,357} = 13.4$ $p = 2.9 \times 10^{-4}$	$\eta_\mathrm{p}^2 = 0.05$ $F_{1,357} = 20.6$ $p = 7.8 \times 10^{-6}$	$\eta_p^2 = 0$ $F_{1,357} = 0.3$ $p = 0.09$	$\eta_p^2 = 0$ $F_{1,357} = 0$ $p = 0.56$
Mixed	$\eta_\mathrm{p}^2 = 0.16$ $F_{1,350} = 67.8$ $p = 3.7 \times 10^{-15}$	$\eta_\mathrm{p}^2 = 0$ $F_{1,350} = 0.1$ $p = 0.74$	$\eta_\mathrm{p}^2 = 0$ $F_{1,350} = 0.9$ $p = 0.34$	$\eta_p^2 = 0.01$ $F_{1,350} = 4.8$ $p = 0.03$	$\eta_p^2 = 0.12$ $F_{1,350} = 47.2$ $p = 2.9 \times 10^{-11}$

To compare the effect of training on various feature-detector parameters compared to parameters of the model trained only using clean calls, we used the F-test as well as the partial eta-squared metric, which approximates the fraction of total variance captured by training. Except for duration and threshold with noisy and mixed training, other parameters were not substantially ($\eta_\mathrm{p}^2 \leq .05$) different relative to clean-call-trained model parameters.

Supplementary Table S3: Effect of noisy and reverberation training on FD properties for guinea pig calls.

Training	Duration	CF	Bandwidth	Kurtosis	Threshold
Noise	$\eta_{\mathrm{p}}^2 = 0$<br>$F_{1,513}$ =3.2<br>$p = 0.74$	$\eta_{\mathrm{partp}}^2 = 0.02$<br>$F_{1,513} = 9.6$<br>$p = 2.0 \times 10^{-3}$	$\eta_{\mathrm{p}}^2 = 0.04$<br>$F_{1,513} = 19.7$<br>$p = 1.1 \times 10^{-5}$	$\eta_{\mathrm{p}}^2 = 0$<br>$F_{1,513} = 0.34$<br>$p = 0.56$	$\eta_{\mathrm{p}}^2 = 0.01$<br>$F_{1,513} = 5.6$<br>$p = .02$
Reverb	$\eta_{\mathrm{p}}^2 = 0.02$<br>$F_{1,567} = 12.4$<br>$p = 4.5 \times 10^{-4}$	$\eta_{\mathrm{p}}^2 = 0$<br>$F_{1,567} = 1.36$<br>$p = 0.24$	$\eta_{\mathrm{p}}^2 = 0$<br>$F_{1,567} = 0.8$<br>$p = 0.37$	$\eta_{\mathrm{p}}^2 = 0$<br>$F_{1,567} = 0.03$<br>$p = 0.86$	$\eta_{\mathrm{p}}^2 = 0$<br>$F_{1,567} = 0.1$<br>$p = 0.71$
Mixed	$\eta_{\mathrm{p}}^2 = 0$<br>$F_{1,617} = 1.1$<br>$p = 0.29$	$\eta_{\mathrm{p}}^2 = 0.02$<br>$F_{1,617} = 9.7$<br>$p = 1.9 \times 10^{-3}$	$\eta_{\mathrm{p}}^2 = 0$<br>$F_{1,617} = 3.9$<br>$p = .048$	$\eta_{\mathrm{p}}^2 = 0$<br>$F_{1,617} = 3.9$<br>$p = 0.047$	$\eta_{\mathrm{p}}^2 = 0.01$<br>$F_{1,617} = 6.4$<br>$p = 0.01$

Same as Supplementary Table S2 but for guinea pig calls. Training did not substantially affect the distribution of any feature-detector parameter ($\eta_{\mathrm{p}}^2 \leq .04$).

Supplementary Table S4: Effect of contrast-gain control and top-down modulation on model performance relative to baseline model.

	Contrast-gain control (CGC)		Top-down modulation		CGC and Top-down	
	Marmoset	Guinea pig	Marmoset	Guinea pig	Marmoset	Guinea pig
Test in noise	$\chi^2(3) = 150.3$ $p < 2.2 \times 10^{-16}$	$\chi^2(4) = 64.5$ $p = 3.2 \times 10^{-13}$	$\chi^2(3) = 144.7$ $p < 2.2 \times 10^{-16}$	$\chi^2(4) = 169.0$ $p < 2.2 \times 10^{-16}$	$\chi^2(3) = 200.8$ $p < 2.2 \times 10^{-16}$	$\chi^2(4) = 128.4$ $p < 2.2 \times 10^{-16}$
Test in Reverb	$\chi^2(3) = 196.3$ $p < 2.2 \times 10^{-16}$	$\chi^2(4) = 93.5$ $p < 2.2 \times 10^{-16}$	$\chi^2(3) = 47.4$ $p = 2.9 \times 10^{-10}$	$\chi^2(4) = 34.4$ $p = 6.1 \times 10^{-7}$	$\chi^2(3) = 211.4$ $p < 2.2 \times 10^{-16}$	$\chi^2(4) = 93.8$ $p < 2.2 \times 10^{-16}$

Both contrast gain control and top-down modulation improved model test performance when operational in isolation as well as simultaneously.
